# Surviving a Dark Age: The Oldest Baleen-Bearing Whales (Cetacea: Chaeomysticeti) of Pacific South America (Lower Miocene, Peru)

**DOI:** 10.3390/life15030452

**Published:** 2025-03-13

**Authors:** Francesco Nobile, Olivier Lambert, Giovanni Bianucci, Eli Amson, Mark Bosselaers, Giulia Bosio, Luca Pellegrino, Elisa Malinverno, Claudio Di Celma, Mario Urbina, Alberto Collareta

**Affiliations:** 1 Dipartimento di Scienze della Terra, University of Pisa, 56126 Pisa, Italy; francesco.nobile@phd.unipi.it (F.N.); giulia.bosio.giulia@gmail.com (G.B.); alberto.collareta@unipi.it (A.C.); 2 D.O. Terre et Histoire de La Vie, Institut Royal Des Sciences Naturelles de Belgique, 1000 Brussels, Belgium; olambert@naturalsciences.be (O.L.); mark.bosselaers@telenet.be (M.B.); 3 Paleontology Department, Staatliches Museum für Naturkunde Stuttgart, 70191 Stuttgart, Germany; eli.amson@smns-bw.de; 4 Dipartimento di Scienze della Terra, University of Turin, 10125 Turin, Italy; lu.pellegrino@unito.it; 5 Dipartimento di Scienze dell’Ambiente e della Terra, University of Milano-Bicocca, 20126 Milan, Italy; elisa.malinverno@unimib.it; 6 Scuola di Scienze e Tecnologie, University of Camerino, 62032 Camerino, Italy; claudio.dicelma@unicam.it; 7 Departamento de Paleontologia de Vertebrados, Museo de Historia Natural—UNMSM, Lima 15072, Peru; mariourbina01@hotmail.com

**Keywords:** Balaenomorpha, baleen whale, Burdigalian, Chilcatay Formation, Early Miocene, East Pisco Basin, Ica Desert, Mysticeti, palaeocetology, Plicogulae

## Abstract

The evolution of baleen whales (Mysticeti) comprises two main phases, namely, (i) a Paleogene phase, which saw the diversification of stem lineages, and (ii) a Neogene phase, dominated by modern-looking, toothless, baleen-bearing forms in the monophyletic group Chaeomysticeti. These two phases are separated by a global turnover event coinciding with a gap—or “dark age”—in the mysticete fossil record. This dark age occurred between 23 and ~18 Ma and is apparently detected worldwide, except in Zealandia. Here, we report on a new mysticete fossil from the Lower Miocene (Burdigalian: ~19.2 Ma) strata of the Chilcatay Formation cropping out at the newly discovered locality of Cerro Tiza (East Pisco Basin, Peru), which represents a limited but precious testament from the last phase of the baleen whale dark age. Two previously mentioned, slightly geologically younger fossils from the same formation are also reappraised herein, revealing the occurrence of at least another baleen whale taxon in the upper Chilcatay strata—one that belongs in the mysticete crown group. Although the Early Miocene remains a problematic time interval for the fossil record of baleen whales, our new results encourage the search for mysticete fossils in the Lower Miocene strata of the East Pisco Basin, whose basin fill preserves a cornucopia of extraordinarily informative marine vertebrate fossils of the Cenozoic age, as well as in coeval deposits worldwide.

## 1. Introduction

Since the passing of the millennium, many aspects of the evolution of the cetacean suborder Mysticeti have been clarified through the description of new fossils. These finds revealed the major skeletal changes accompanying the emergence of the modern lineages of baleen-bearing whales starting from a diverse stock of early toothed forms (e.g., [1,2,3]). Thus, the evolutionary history of mysticetes can be summarized in a Paleogene phase that saw the diversification of toothed families (e.g., aetiocetids and mammalodontids) as well as the radiation of the earliest baleen-bearing forms (i.e., the eomysticetids) (e.g., [4,5,6]) and a Neogene phase that saw the rise of the modern baleen whales in the crown-group Mysticeti (e.g., [1,3]). Separating these two phases is a global turnover event coinciding with a global gap (or “dark age”) in the mysticete fossil record [5,7]. This dark age lasted between 23 and ~18 Ma and is apparent worldwide, except in Zealandia [5,7]. Although this timespan presents challenging conditions for the discovery of new baleen whale fossils (e.g., the lack of cetacean assemblages from offshore settings, which may have been the preferred habitats of the toothless mysticetes; [7]), the mysticete dark age still has its witnesses. The present paper aims at providing a short report on a new baleen whale fossil from lower Burdigalian (19–18.7 Ma) sediments of the Chilcatay Formation exposed at Cerro Tiza, a newly investigated locality of the renowned Pisco Lagerstätte of Peru [8,9,10,11,12,13]. The same formation already yielded two mysticete specimens [14] that are also briefly reappraised herein.

## 2. Materials and Methods

The new specimen from Cerro Tiza was discovered in February 2024 and assigned the field number CTZ 02. It was then collected and deposited in the Museo de Historia Natural de la Universidad Nacional Mayor de San Marcos (=MUSM, Lima, Peru) under the catalogue number MUSM 5250. The two mysticete specimens mentioned by [14] as originating from the Chilcatay Formation were left in the field, but they are nonetheless unambiguously identified by the field numbers ZM 152 (a fairly complete skull) and ZM 98 (a dentary) (see [14]).

The systematic affinities of the three studied specimens were assessed through a detailed review of the relevant palaeontological literature [1,3,15,16,17,18,19,20,21,22,23,24,25,26,27,28,29,30,31,32,33,34,35,36,37,38,39,40,41,42,43,44,45,46,47,48,49,50,51,52,53,54,55,56].

Photographs of all the specimens were taken using a Sony α6000 mirrorless camera equipped with a 16–50 mm f/3.5–5.6 lens. The focal length was kept constant while working on each specimen. A scaled 3D model of CTZ 02 was acquired using the structured-light scanner EinScan Pro HD 2020. It is freely available as Supplementary Materials File S1. In addition, the textured 3D model of ZM 152 was processed in the Agisoft Metashape software, 1.7.6 version, by masking and aligning 86 photographs. This photogrammetric model can be downloaded as Supplementary Materials File S2. Digital renderings of the textured photogrammetric 3D model of ZM 152 were obtained with Blender 4.1.

Sediment samples were collected at the find locality of CTZ 02, along a local section as well as around the fossil specimen, for biostratigraphic analyses. The samples were prepared with the standard smear slide method using a cover slip and Norland mounting medium [57]. All the samples were analysed with a Olympus BX50 light microscope at 1000× with immersion oil at the Department of Earth and Environmental Sciences of the University of Milano Bicocca. First Occurrences (hereinafter: FOs) and Last Occurrences (hereinafter: LOs) are as per the Supporting Information of [58], which provides recalibrations to the recent timescale by [59] (note that most of their bioevents were originally calibrated for the North and Equatorial Pacific).

Data from [8,11,12,14] were used for calculating the relative abundance of marine tetrapods along the Miocene succession of the East Pisco Basin.

## 3. Geological Setting

**Figure 1:**
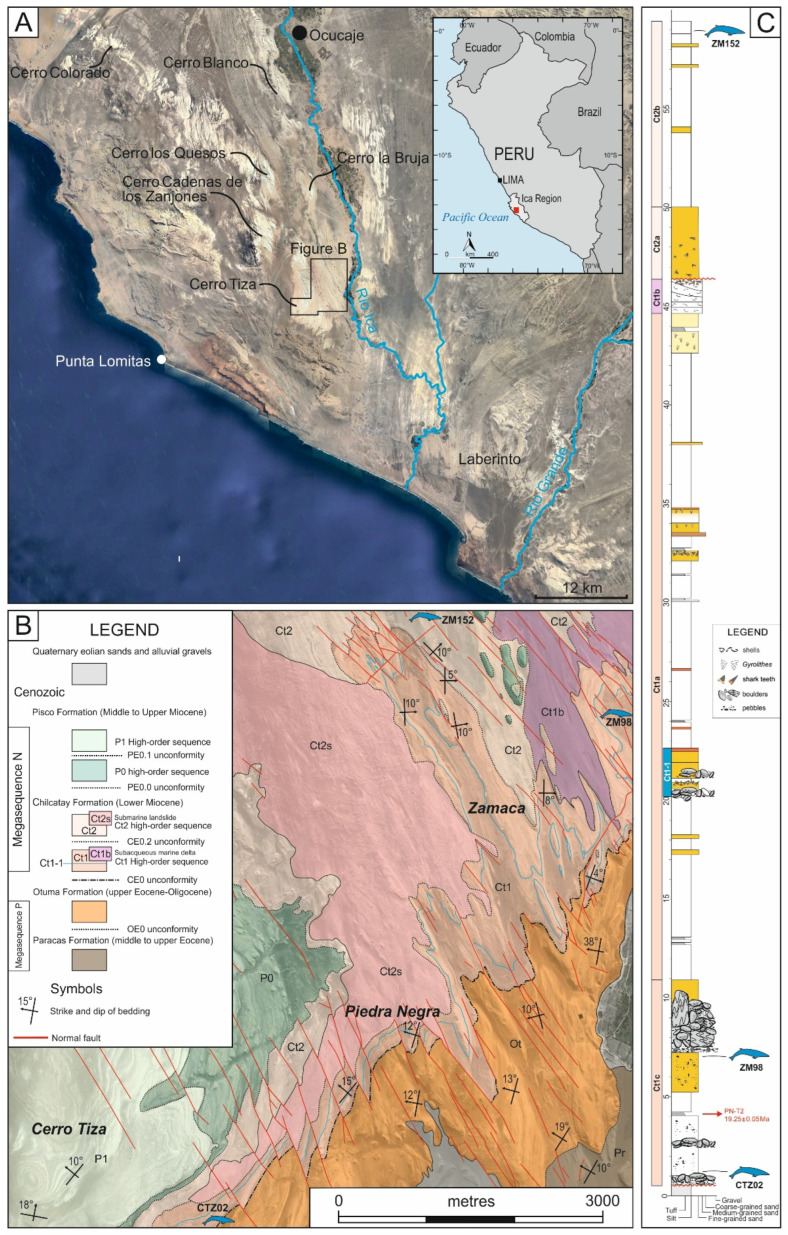
Geographic and geological setting. (**A**) Satellite view of the Ica Desert, showing the location of the Cerro Tiza–Zamaca–Piedra Negra area as well as of the main fossiliferous “cerros”. (**B**) Schematic geological map of the Cerro Tiza–Zamaca–Piedra Negra area, showing the find sites of the three mysticete specimens dealt with herein. (**C**) Composite stratigraphic log of the Chilcatay Formation in the Cerro Tiza–Zamaca–Piedra Negra area, showing the stratigraphic position of the three mysticete specimens in question (vertical scale is in metres).

Bounded by the Coastal Batholith to the east and the Outer Shelf High to the west, the East Pisco Basin is an Andean forearc basin whose essentially marine sedimentary fill is almost entirely exposed subaerially along the southern Peruvian coast, first and foremost in the Ica Valley [13,60,61] (Figure 1A). These sediments have recently been divided into two megasequences (Figure 1B), namely, Megasequence P (=Paleogene) and Megasequence N (=Neogene) [62]. Megasequence P includes the middle to upper Eocene Paracas Formation [63,64] and the upper Eocene to lower Oligocene Otuma Formation [64,65]. The Paracas sequence consists of medium- to coarse-grained bioclastic sandstones that grade upward into a monotonous succession of green-weathering siltstones, representing deposition in shoreface and relatively deeper inner- to outer-shelf settings, respectively [62]. The Otuma sequence consists of a concretionary, fine- to medium-grained, sandstone package overlain by finely laminated siltstones [62]. Megasequence N includes two Lower Miocene (allo)formations, namely, the Tunga Formation (which is likely not exposed in the Ica Valley proper; [66]) and Chilcatay Formation [14,67,68] and the Middle to Upper Miocene Pisco Formation [62,69]. The Chilcatay sequences comprise coarse-grained bioclastic sandstones interbedded with conglomerates enriched in basement clasts, which pass stratigraphically upward into laminated siltstones with sand- and gravel-rich intercalations, interpreted as a subaqueous marine delta [62]. The youngest Pisco Formation is characterized by fining-upward units that consists of sandstones at the base (onshore) and diatomaceous siltstones at the top (offshore), representing three transgressive cycles.

The Chilcatay Formation, which yielded the mysticete specimens dealt with herein, has been further divided into three sequences or allomembers, namely, Ct0, a poorly known stratal package that was first described in the southern Laberinto area (20–19.5 Ma, [66,70]) and has been subsequently recognized at the Media Luna locality (21.8–20.1 Ma, [71]) but not at our study area of Cerro Tiza; Ct1 (19.2–18.4 Ma), which is known from the areas of Ullujaya and Zamaca and includes three facies associations, referred to as Ct1c, Ct1a, and Ct1b in ascending stratigraphic order [14,68,71,72]; and Ct2 (18.4–18.0 Ma), including two facies associations, referred to as Ct2a and Ct2b in ascending stratigraphic order [14,68,71].

Both the newly discovered mysticete specimen, CTZ 02, which was found at Cerro Tiza (geographic coordinates: 14°40′10.3″ S–75°40′16.6″ W), and the previously reported specimen ZM 98, which was found at Zamaca, originate from the basal Ct1c facies association of the Chilcatay Formation and, specifically, from below the “Piedra Negra (=PN) oyster bed”, corresponding to the Ct1-1 marker bed, which in turn can be traced laterally from one locality to the other (Figure 1B,C). This horizon has been assigned an age of 19.1–18.7 Ma based on Strontium Isotope Stratigraphy [71,72]. Dating of the underlying volcanic ash layer PN-T2 yielded an age of 19.25 ± 0.05 Ma [73], which is supported by biostratigraphic data that assign this interval to the silicoflagellate *Naviculopsis ponticula* zone of [74], dated between ~19 Ma and ~18 Ma [75].

ZM 98 occurs ~14 m below the Ct1-1 marker bed ([14]: main map), resulting in an age between 19.25 ± 0.05 Ma (corresponding to the PN-T2 ash layer) and 19.1–18.7 Ma (corresponding to Ct1-1). On the other hand, the horizon that yielded the Cerro Tiza mysticete is located 6 m below, occurring just above the CE0.1 unconformity that marks the base of the Chilcatay Formation, thus being likely stratigraphically lower than the PN-T2 ash layer [73]. Sediments from the strata in which the new specimen were found contain a diatom assemblage typical of Ct1 (including *Thalassiosira fraga*, FO: 20.4 Ma, LO: 16.3 Ma; *Raphidodiscus marylandicus*, FO: 23.9 Ma, LO: 16.5 Ma; and *Cavitatus jouseanus*, FO: 28.7 Ma, LO: 6.8 Ma) as well as the silicoflagellate species *Naviculopsis ponticula* (FO ~19 Ma, LO ~18 Ma) and *Naviculopsis stradneri*. The latter has been reported by [76] to disappear slightly before the appearance of *N. ponticula*, such that their seeming co-occurrence further supports an age slightly older than 19 Ma for the basal interval of Ct1c, in general, and for CTZ 02, in particular. Considering the concurrent biostratigraphic and radiometric data, a conservative age of ~19.2 is assumed herein for CTZ 02.

The third mysticete specimen dealt with in this paper, namely, ZM 152 from Zamaca, comes from the Ct2b sequence, which has been dated between 18.4 and 18.0 Ma [68,71] (Figure 1B,C).

## 4. The New Mysticete Skull from Cerro Tiza

**Figure 2:**
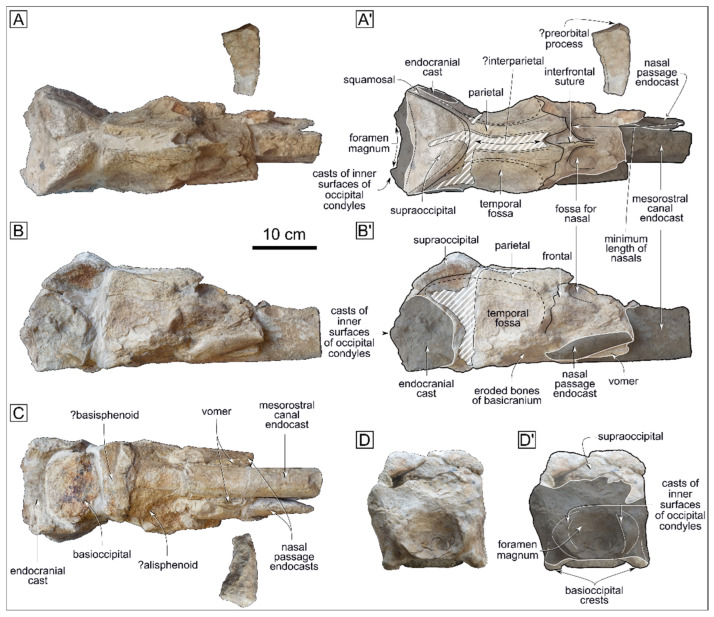
The Cerro Tiza mysticete specimen, CTZ 02, in dorsal view ((**A**), photograph; (**A’**), interpretative line drawing), right lateral view ((**B**), photograph; (**B’**), interpretative line drawing), ventral view ((**C**), photograph), and posterior view ((**D**), photograph; (**D’**), interpretative line drawing). White-dashed areas indicate glued areas, whereas dark-shaded areas indicate sediment.

The Cerro Tiza mysticete specimen, CTZ 02, consists of a partial neurocranium, measuring 44 cm in total preserved length, which was found associated with a small fragment of bone that seemingly preserves the anterolateral corner of the supraorbital process of the frontal, including the preorbital process (Figure 2A–C). The preserved neurocranial bones include parts of the frontals anterior to the parietals, shreds of the vomer, the parietals, the incomplete left squamosal, possibly the eroded alisphenoids and basisphenoid, the supraoccipital, and an eroded basioccipital (Figure 2A–C). Additionally, the specimen preserves most of the endocranial cast, including casts of the inner surfaces of the occipital condyles and foramen magnum (Figure 2B–D), and endocasts of the mesorostral canal as well as of the nasal passages. Judging from the incomplete fusion of various sutures, including the interfrontal and medial parietal sutures on the dorsal skull surface (Figure 2A,A’; [77,78]), these remains may belong to a juvenile.

Based on the observation of a rather clear posterior edge of the fossa for the left nasal on the dorsal surface of the frontal and considering the anteriormost extent of the left nasal passage endocast (Figure 2A,A’) as well as the fact that the preserved endocasts do not bend dorsomedially, the nasals would have been very long anteroposteriorly (at least 175 mm and most likely longer); the rationale for this inference being that the nasals roof the nasal passages dorsally rather than laterally in mysticetes.

The most striking feature of the Cerro Tiza specimen is the relatively long (ca. 115 mm) dorsal exposure of the parietals in the intertemporal region, which is roughly as long as the dorsal exposure of the occipital along the sagittal plane (see description below), but still slightly shorter than the reconstructed length of the nasals. When viewed dorsally, most of the frontoparietal suture is convex anteriorly but slightly interdigitating through a W-shaped median part. The anteroventral corner of the parietal turns anterolaterally, thus hinting at the posterodorsomedial onset of the supraorbital process. When viewed dorsally, the two parietals do not contact each other, being rather divided medially by a rather deep, anteroposteriorly elongated empty space. As the parietals do not show any sign of dislocation and as the medial sutural surfaces of the parietals are unfused and do not match one another, this empty space may have been home to a narrow, anteroposteriorly long exposure of the interparietal (possibly the anterior median interparietal; see [78]). Given this condition and considering the flat dorsolateral surfaces of the parietals in the intertemporal region, there is no indication whatever of the presence of a sagittal crest. The lateral walls of the parietals are steep and define a stout intertemporal constriction that is especially evident in the dorsal view.

The vertex is placed along the nuchal crest, which is shaped as a blunt triangle in the dorsal view. The poorly telescoped occipital shield bears no noticeable external occipital crest, but the medial part of the shield is somewhat damaged (Figure 2D,D’). In the lateral view, the occipital slopes at a relatively steep angle (ca. 60°) with respect to the horizontal plane (Figure 2B,B’).

In the posterior view, the foramen magnum is seemingly wide, whereas the basioccipital crests project ventrolaterally, being slightly wider than the condyles (Figure 2D,D’).

CTZ 02 displays an eroded, poorly informative ventral surface, in which the worn-out vomer, alisphenoids, basisphenoid, and basioccipital are largely preserved (Figure 2C).

## 5. Other Baleen Whales from the Chilcatay Formation

### 5.1. ZM 98

**Figure 3:**
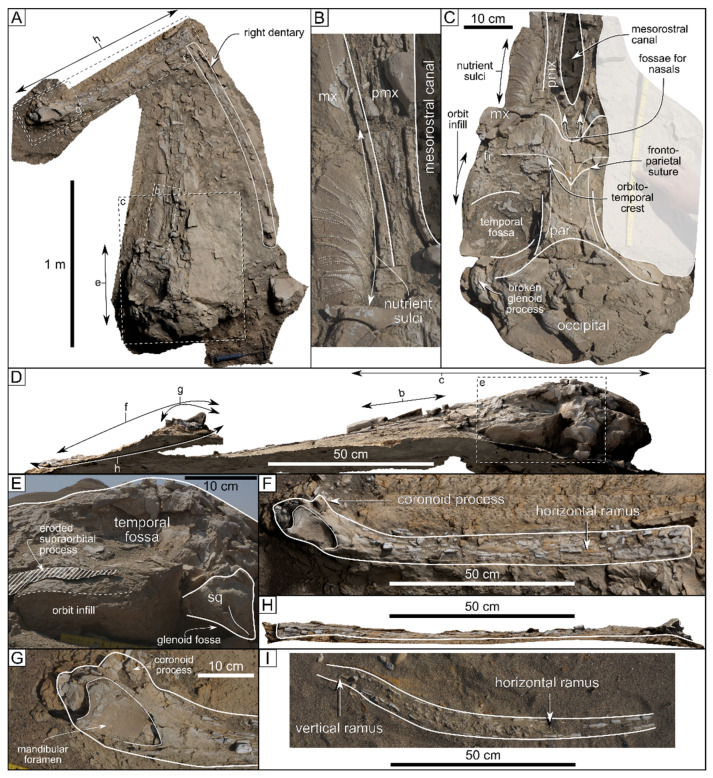
The Zamaca mysticete specimens ZM 152 (**A**–**H**) and ZM 98 (**I**). (**A**) digital rendering of the textured model of ZM 152, showing the cranium in dorsal view, left dentary in medial view, and right dentary in dorsal view. (**B**) Photograph of ZM 152, close-up of the external imprint of the nutrient sulci on the left side of the palate. (**C**) Photograph of ZM 152, interpretative line drawing of the neurocranium in dorsal view. (**D**) Digital rendering of the textured model of ZM 152, cranium in left lateral view. (**E**) Photograph of ZM 152, close-up of the temporal fossa and squamosal in left lateral view. (**F**) Digital rendering of the textured model of ZM 152, close-up of the left dentary in medial view. (**G**) Photograph of ZM 152, close-up of the left mandibular condyle and mandibular foramen. (**H**) Digital rendering of the textured model of ZM 152, close-up of the left dentary in dorsal view. (**I**) Photograph of ZM 98, badly damaged dentary preserved in either dorsal or ventral view. Abbreviations: fr = frontal; mx = maxilla; par = parietal; pmx = premaxilla; sq = squamosal.

ZM 98 ([14] figure 5D) is a badly damaged dentary preserved in either dorsal or ventral disposition (Figure 3I). Erosion in the present-day desert environment has led to the destruction of the bone down to the sediment-filled mandibular canal. The arc length of ZM 98 is 93 cm. The horizontal ramus is evenly bowed laterally, whereas what remains of the vertical ramus appears to be oriented at an angle. As the ZM 152 left dentary is preserved in medial disposition and the right one is damaged (see description below), comparisons between these two specimens are hindered.

### 5.2. ZM 152

The stratigraphically youngest mysticete find from the Chilcatay Formation (ZM 152; [14]: figure 5c) consists of a fairly complete cranium lying dorsal side up and the two dentaries (Figure 3A). The cranium preserves the fragmentary maxillae and premaxillae, parts of the supraorbital process of the left frontal, parts of the glenoid process of the left squamosal, the vertex region, and most of the occipital shield. What is left of the rostral bones allows for the reconstruction of the general outline of the mesorostral canal but not the lateral outline of the rostrum (except for the very base thereof). In addition, the palatal surface of the left maxilla is also preserved in the form of a partial external imprint in the hardened entombing sediment (Figure 3B).

The imprint of the proximal palatal surface of the left maxilla reveals the presence of nutrient sulci (Figure 3B,C) anterior to the rostrum base and lateral to the mesorostral canal. These sulci are strongly bent anterolaterally. We interpret them as the sulci for the superior alveolar arteries [79]. The density of the nutrient sulci, which is very high close to the rostrum base, appears to decrease anteriorly but not to the point of disappearing, entering as they do the apical half of the rostrum (the palatal surface of the anterior rostrum is not preserved not even as an external imprint). The premaxilla–maxilla suture is also preserved along at least the posterior third of the rostrum (Figure 3B).

Judging from the posterior outline of the dorsal opening of the mesorostral canal (Figure 3C), the nasals should have been relatively short and certainly much more so than in CTZ 02.

When viewed laterally, the supraorbital process of the frontal descends to a distinctly low dorsoventral level compared with the vertex (Figure 3D). It bears a gently curved orbitotemporal crest, which starts from the frontoparietal suture and runs transversely across the preserved portion of the supraorbital process. The posterior border of the supraorbital process projects laterally and slightly posteriorly. The frontoparietal suture is concave in its median part (Figure 3C) due to the posterodorsal corners of the frontals wedging in between the parietals.

Due to the limited anterior telescoping of the occipital shield (see description below), the dorsal exposure of the parietals in the intertemporal region is moderately long. Although the parietals contact each other dorsomedially, thus differing from the condition observed in CTZ 02, they show no signs of a sagittal crest (which, however, may have been obliterated by erosion in the present-day desert environment). When viewed dorsally, the temporal fossa is roughly oval and slightly transversely wider than long (Figure 3C).

The partly preserved glenoid process includes a posteroventrally directed postglenoid process of the squamosal (Figure 3E). When viewed laterally, the glenoid fossa displays a regularly curved outline (Figure 3E).

The occipital shield slopes much less steeply (ca. 40° with respect to the horizontal plane) than observed in CTZ 02 (Figure 3D). Its anterior border is broadly rounded. The dorsal exposure of the occipital along the sagittal plane is distinctly longer than in CTZ 02, much more so than the dorsal exposure of the parietals in the intertemporal region (Figure 3C).

The right dentary is poorly preserved but seemingly complete anteroposteriorly (Figure 3A). Together with what is left of the premaxillae close to the mesorostral canal, it allows for estimating the condylobasal length of ZM 152 around 160 cm, with the neurocranium accounting for about 27–29% of the total skull length.

The left mandibular ramus is better preserved than its right antimere (Figure 3A,F). It lies with its medial side up (Figure 3F–H) and preserves most of the horizontal ramus, the base of the coronoid process, and the mandibular foramen (Figure 3G). The onset of the dorsal elevation of the coronoid process is observed just above the overall well-preserved mandibular foramen, which is large and triangular in shape (Figure 3F,G).

## 6. Systematic Affinities of the Chilcatay Mysticetes

### 6.1. Preliminary Remarks

Consensus on the phylogeny of the baleen-bearing whales (Chaeomysticeti) is still wanting, as the continuous discovery of new fossils and the inclusion or exclusion of specific taxa in different phylogenetic analyses has led different authors to propose several alternative phylogenetic reconstructions (e.g., [1,3,4,6,40,45,48,54,80,81]). Nevertheless, most recent phylogenetic reconstructions (including, as far as the living species are concerned, those based solely on genetic/genomic data; [82]) have consistently recovered the following groups: (i) the extinct family Eomysticetidae, representing the earliest diverging chaeomysticete lineage; (ii) Balaenidae, the basalmost family of crown mysticetes (=Balaenomorpha sensu [31]), which includes the extant genera *Balaena* and *Eubalaena* of right and bowhead whales, and many extinct forms; (iii) Cetotheriidae, a seemingly more crownward mysticete clade that may include the extant pygmy right whale, the “neobalaenid” *Caperea marginata* ([83], but see also [84]); (iv) Balaenopteroidea, including Balaenopteridae (rorquals and humpback whales) and Eschrichtiidae (grey whales, sometimes recognized as a subfamily of the balaenopterids), and a number of fossil forms.

Cetotheriids and balaenopteroids are generally seen as comprising the monophyletic group Plicogulae (sensu [85] = Thalassotherii sensu [39]; see [6], for the synonymy of the two terms) along with a number of archaic-looking forms. Within this grouping, several phylogenies have recognized the existence of a clade of “basal plicogulans” (=“basal thalassotherians”) that includes *Parietobalaena* and *Diorocetus* and often also *Isanacetus*, *Atlanticetus patulus*, and *Pelocetus calvertensis* among other forms [1,3,40,54,80,81]. More recently, [6] has proposed the existence of an even more basal “*Toipahautea*-to-*Mauicetus* grade” of toothless mysticetes—one that may represent either the sister group of Balaenomorpha or an early-diverging stock of crown mysticetes leading to the plicogulans sensu stricto (Balaenopteroidea + Cetotheriidae; [6]) through the late Oligocene genera *Whakakai*, *Toipahautea*, *Horopeta*, and *Mauicetus*.

In the paragraphs that follow, we will mostly refer to the aforementioned groupings to discuss the systematic affinities of the Chilcatay mysticetes.

### 6.2. Affinities of CTZ 02

CTZ 02 differs from the Paleogene forms of toothed mysticetes and from some archaic chaeomysticetes (including all eomysticetids but *Tohoraata raekohao* and some basal plicogulans) as the arrangement of the parietals in the intertemporal region is at odds with the presence of a sagittal crest, which in turn occurs in the aforementioned taxa [3]. CTZ 02 further differs from the eomysticetids as the members of this family display a frontoparietal suture that is well posterior to the onset of the supraorbital process as well as a generally slenderer intertemporal constriction [29,37,43,80]. Nonetheless, CTZ 02 shares with the eomysticetids several plesiomorphies such as strongly elongated nasals [2,42,86]; the long dorsal exposure of the parietals in the intertemporal region [3,46,80]; a slightly interdigitating, anteriorly convex frontoparietal suture; and an occipital shield that is only poorly telescoped [2]. These characters further associate CTZ 02 with *Horopeta* [46], a member of the “*Toipahautea*-to-*Mauicetus* grade” [6].

A referral of CTZ 02 to some other group of crown mysticetes (including the “basal plicogulans”) is discouraged (and even precluded as far as the extant families are concerned) by the observation of a poorly telescoped supraoccipital, this bone being typically more distinctly projecting onto the parietals in balaenomorphs [2], as well as by the remarkable reconstructed length of the nasals [86]. That said, some phenetic similarities, such as the involvement of the anteroventral corner of the parietal in the anterior broadening of the intertemporal constriction (Felix Marx, pers. comm., 2024), can be pointed out with, e.g., *Aglaocetus moreni*, a phylogenetically labile chaeomysticete taxon that has been alternatively retrieved as a stem chaeomysticete (e.g., [54]) or as a crown-group mysticete at the base of the cetotheriid clade [3].

In conclusion, we identify CTZ 02 as an archaic chaeomysticete and possibly one close to the late Oligocene genus *Horopeta*. Whether CTZ 02 is a late-diverging stem mysticete or an early-branching crown mysticete remains unclear to date.

### 6.3. Affinities of ZM 98

Systematically framing ZM 98 is difficult due to its substantial incompleteness. However, we identify this specimen as a member of the chaeomysticete clade crownward of Eomysticetidae based on the presence of a horizontal ramus that is evenly bowed laterally [1].

### 6.4. Affinities of ZM 152

ZM 152 differs from the Paleogene forms of toothed mysticetes by featuring a dorsoventrally thin, mediolaterally wide rostrum that is distinctly longer than the neurocranium [1,2,3]. It further differs from all toothed mysticetes but the aetiocetids by the nasals being shortened (i.e., shorter than the dorsal exposure of frontals along the sagittal plane) as well as by displaying osteological correlatives of the baleen in the form of palatal nutrient sulci [87].

ZM 152 differs from the eomysticetids by possessing well-developed palatal nutrient sulci that occur throughout the preserved length of the rostrum (a typical character of Balaenomorpha; [2]); shorter nasals [2,42,86]; a smaller, anteroposteriorly shorter temporal fossa that is wider than it is long [2,3,80]; a stouter intertemporal constriction [1,2]; and a shorter dorsal exposure of the parietals in the intertemporal region, these bones being shorter than the dorsal exposure of the supraoccipital along the sagittal plane.

Comparisons with members of the so-called “*Toipahautea*-to-*Mauicetus* grade” suggest that ZM 152 is representative of a more advanced skull architecture—one that features an anteriorly concave frontoparietal suture and a more telescoped supraoccipital (but see the rather similar condition in *Whakakai*).

ZM 152 strongly differs from Balaenidae by featuring a rostrum that is sub-rectilinear in the lateral view; a supraorbital process that is broad and gently sloping; parietals that are well-exposed dorsally in the intertemporal region; an occipital shield that is only moderately ascending; and a coronoid process of the dentary that is well developed.

ZM 152 resembles the basal plicogulans and “basal Cetotheriidae” (sensu [3]—namely, *Aglaocetus*, *Cophocetus,* and *Titanocetus*) in several anatomical traits, including a moderately long dorsal exposure of the parietals in the intertemporal region (length of the parietals and occipital is subequal along the sagittal plane) and an oval temporal fossa. The frontoparietal suture of ZM 152 is anteriorly concave, which is reminiscent of the condition observed in some cetotheriids [80].

ZM 152 differs from the advanced plicogulans (including Cetotheriidae sensu stricto and Balaenopteroidea) as well as from the “neobalaenids” (whose referral to Plicogulae is debated at present) by retaining a moderately long dorsal exposure of the parietals in the intertemporal region, a limited degree of telescoping of the supraoccipital, and a large mandibular foramen.

In conclusion, ZM 152 can be positively placed within the crown mysticetes and possibly among the basal plicogulans.

## 7. Discussion and Concluding Remarks

Until recently, the South American Lower Miocene record of Mysticeti was essentially limited to *Morenocetus parvus*, *Protororqualus dyticus* (as per the combination proposed by [88]), and *Aglaocetus moreni*, all of which are part of the celebrated marine vertebrate assemblage of the Gaiman Formation of Atlantic Patagonia, Argentina [15,16,50], whose probable age is Burdigalian [89]. These three genera and species are currently regarded as early representatives of just as many groups of crown mysticetes, namely, Balaenoidea, Balaenopteroidea, and Cetotheriidae, respectively [3,50,88]; but see, e.g., [54] for a different interpretation of the affinities of *A. moreni*). Thus, the South American record highlights the importance of the Early Miocene time span for the emergence and early evolutionary history of the major modern mysticete clades. Paradoxically, these Early Miocene originations are only hinted at by just a handful of systematically informative records worldwide.

Given its early Burdigalian (~19.2 Ma) age, the Cerro Tiza specimen is the oldest chaeomysticete known to date from the southeastern Pacific. It comes as some surprise that this specimen is seemingly more reminiscent of Oligocene forms such as *Horopeta* than of other Miocene chaeomysticetes. ZM 152, another informative chaeomysticete find from the upper Chilcatay strata (18.4–18.0 Ma), is in turn representative of a more derived cranial architecture—one that finds correspondences in some Miocene early plicogulans. With the addition of a poorly preserved mandible (ZM 98), these two specimens represent the only finds of Mysticeti from the otherwise odontocete-rich Lower Miocene deposits of the East Pisco Basin ([90], and references therein), as well as the oldest after the Priabonian Eocene toothed form *Mystacodon selenensis*, which in turn is regarded as the oldest representative of the total group Mysticeti ([75]; but see also [81]).

**Figure 4:**
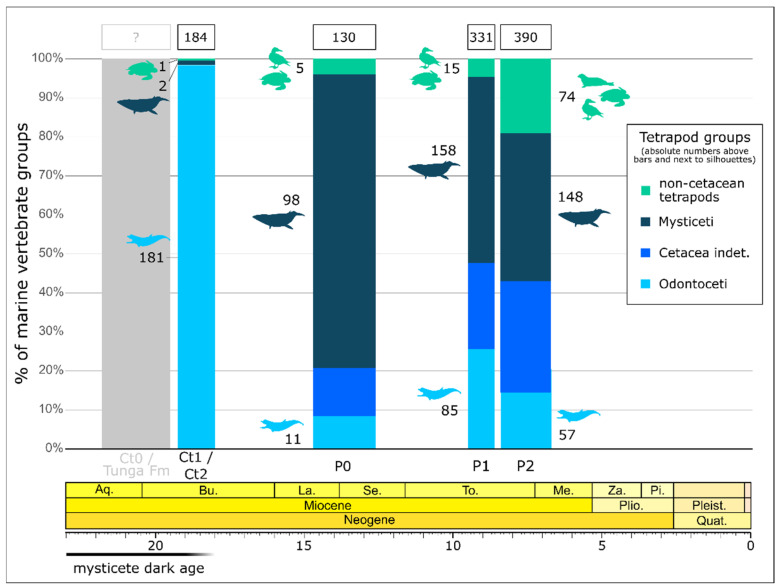
Time-calibrated stacked barplot showing the absolute and relative abundances of marine tetrapods (excluding indeterminate cetaceans) across well-investigated localities of the Chilcatay Formation (Ullujaya and Zamaca; data from [8,14]) and Pisco Formation (sixteen different localities; data from [11,12]). Ct0, Ct1, Ct2, P0, P1, and P2 refer to different sequences or allomembers that comprise the Chilcatay (Ct0–2) and Pisco (P0–2) stratal packages (see [71]). The Tunga Formation was recently described by [66]. Note that data from the newly investigated locality of Cerro Tiza, whose Ct1 strata yielded the mysticete specimen CTZ 02, are not included herein due to incomplete prospecting for fossil vertebrates. The same applies to the Tunga and Ct0 strata, for which no fossil tetrapod censuses have been performed to date (hence the question mark in the figure). Silhouettes of the macroraptorial sperm whale *Acrophyseter deinodon*, the minke whale *Balaenoptera acutorostrata*, the spotted seal *Phoca largha*, the Nazca booby *Sula granti,* and the green sea turtle *Chelonia mydas* were taken from PhyloPic. The chronostratigraphic scale is after [91] (updated in 2023).

An examination of the taxonomic composition of the cetacean assemblages from Lower, Middle, and Upper Miocene strata at well-prospected localities of the Ica Desert reveals that mysticetes are consistently more abundantly represented than odontocetes in the Langhian–Serravallian (P0), Tortonian (P1), and Tortonian–Serravallian (P2) deposits of the Pisco Formation, but they only account for about 1% of the cetacean finds from the Burdigalian (Ct1 and Ct2) strata of the Chilcatay Formation (Figure 4). Thus, in the East Pisco Basin, the Early Miocene mysticete dark age is made evident by the overly scanty presence of baleen whales versus a general abundance of toothed whales around 19–18 Ma (see also [8,14]). The lack of mysticetes in the Burdigalian beds of Ct1 and Ct2 is even more striking as the baleen-bearing whales are overwhelmingly dominant in the overlying P0 strata, in which they account for as much as 90% of the cetacean specimens identifiable at the suborder level (Figure 4). That said, regardless of the paucity of baleen whale remains throughout the best-investigated exposures of the Chilcatay Formation, our discovery of a rather informative specimen at the newly prospected locality of Cerro Tiza encourages the search for Lower Miocene mysticete fossils in the Ica Desert.

Even more elusive than the Burdigalian mysticetes are those from the preceding Aquitanian stage [5]. This is partly due to the fact that cetacean-bearing deposits dating back to the Aquitanian are rare worldwide [7] and are essentially limited to the Libàno Sandstone of northern Italy [92,93,94,95,96]; the Clallam Formation of Washington State, USA [97,98,99,100]; and, possibly, the Nye Mudstone of Oregon [101,102,103,104] and the Belgrade Formation of North Carolina [105,106,107].

It is interesting to note that two Lower Miocene units older than Ct1 have recently been identified at remote localities of the Ica Desert, namely, (1) the basal Ct0 sequence of the Chilcatay Formation, which appears to have deposited not later than the early Burdigalian, and (2) the underlying Tunga Formation, for which an Aquitanian age has been proposed (Figure 4). Although the latter unit appears to be characterized by a remarkable paucity of cetacean fossils [66], both the aforementioned stratal packages are strongly understudied to date, and as such, are well worth additional prospecting.

## Data Availability

The data presented in this study are available in the Supplementary Materials.

## Supplementary Materials

The following supporting information can be downloaded at https://www.mdpi.com/article/10.3390/life15030452/s1: File S1: Scaled and textured 3D model of CTZ 02 (OBJ, MTL, and JPG files); File S2: Scaled and textured 3D model of ZM 152 (OBJ, MTL, and JPG files).
