# Supplementary figures and images for: Surviving a Dark Age: The Oldest Baleen-Bearing Whales (Cetacea: Chaeomysticeti) of Pacific South America (Lower Miocene, Peru)
Source: Life (Basel). 2025 Mar 13;15(3):452. doi: 10.3390/life15030452 (PMC11944254; doi:10.3390/life15030452)

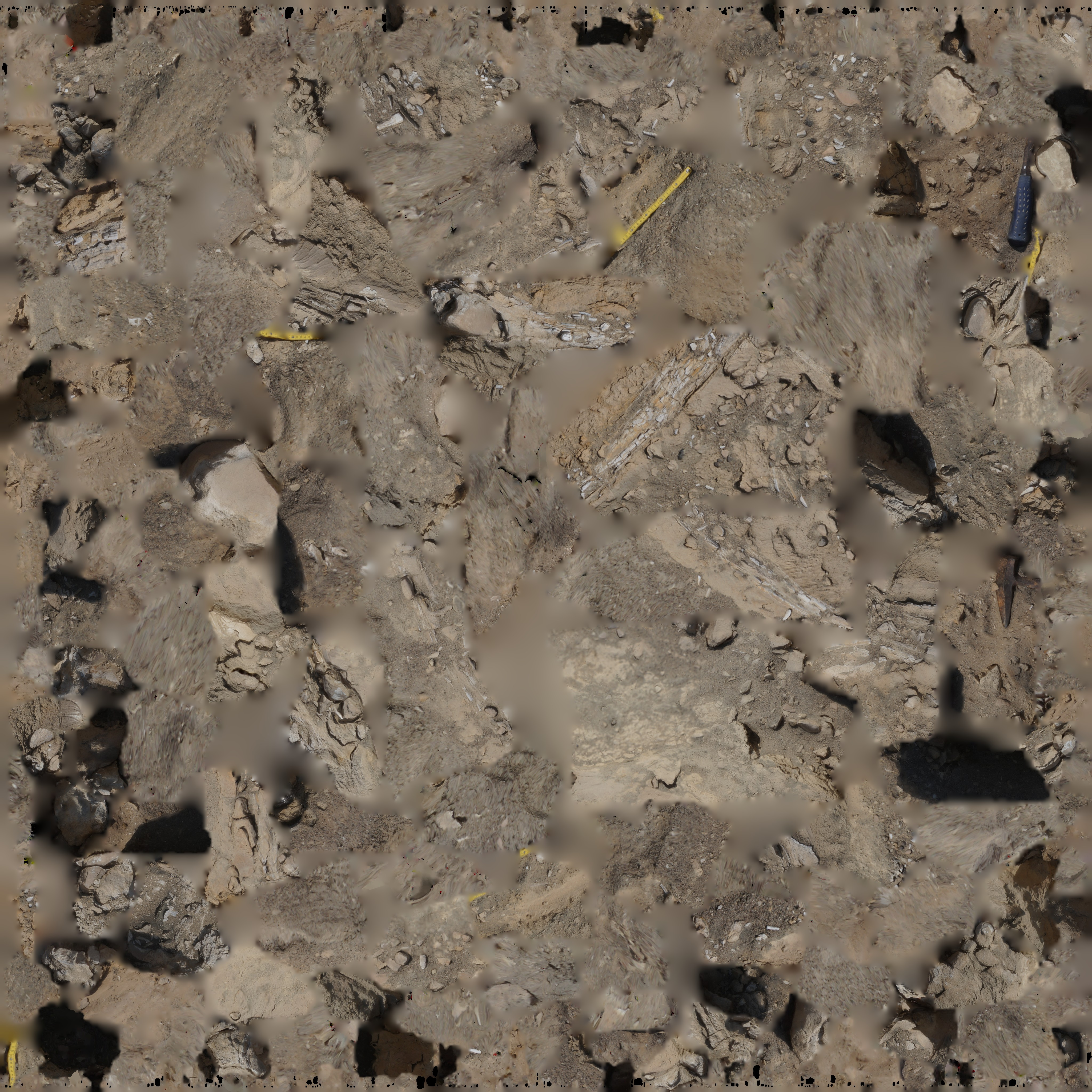

Supplement: Supplementary file 1 [file life-15-00452-s001.zip › S2_ZM152/ZM152_S.png]

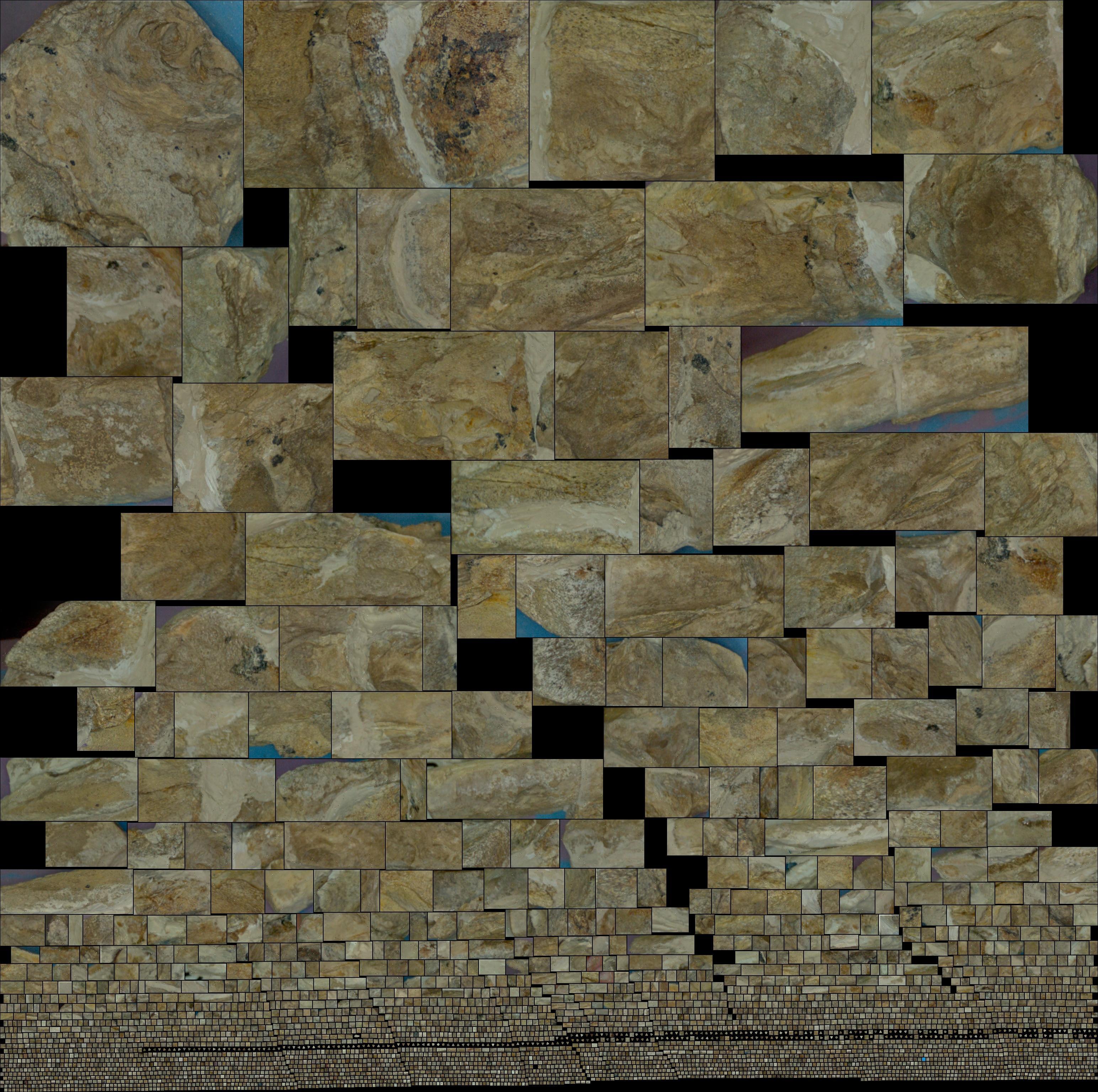

Supplement: Supplementary file 1 [file life-15-00452-s001.zip › S1_CTZ02/CTZ02.jpg]
